# Reductive Synthesis of Stable, Polysaccharide in Situ-Modified Gold Nanoparticles Using Disulfide Cross-Linked Alginate

**DOI:** 10.3390/molecules30244750

**Published:** 2025-12-12

**Authors:** Lyudmila V. Parfenova, Eliza I. Alibaeva, Guzel U. Gil’fanova, Zulfiya R. Galimshina, Ekaterina S. Mescheryakova, Leonard M. Khalilov, Semen N. Sergeev, Nikita V. Penkov, Baoqiang Li

**Affiliations:** 1 Institute of Petrochemistry and Catalysis, Ufa Federal Research Center, Russian Academy of Sciences, Prospekt Oktyabrya, 141, 450075 Ufa, Russia; 2 Department of Materials Science and Physics of Metals, Institute of Technology and Materials, Ufa University of Science and Technology, 12 Karl Marx Street, 450008 Ufa, Russia; 3 Institute of Cell Biophysics of the Russian Academy of Sciences, Federal Research Center “Pushchino Scientific Center for Biological Research of the Russian Academy of Sciences”, Institutskaya 3, 142290 Pushchino, Russia; 4 State Key Laboratory of Urban Water Resource and Environment, Institute for Advanced Ceramics, Harbin Institute of Technology, Harbin 150001, China

**Keywords:** Au nanoparticles, alginic acid, in situ modified with alginate, size stability

## Abstract

Gold nanoparticles (AuNPs) are promising for biomedical applications, but their synthesis often requires toxic reagents. “Green” methods utilizing biopolymers offer a sustainable alternative. This study presents a novel synthesis of stable gold nanoparticles using a disulfide-crosslinked derivative of alginic acid (AA–S–S–AA) as both a reducing agent and stabilizer. The S–S-cross-linked alginate was synthesized with a degree of substitution of ~4.2% and reacted with HAuCl_4_ in water at room temperature for just 10 min to give stable and polysaccharide in situ modified gold nanoparticles (AA-AuNPs). The resulting AA-AuNPs were characterized by a surface plasmon resonance peak at 539 nm and exhibited good colloidal stability over 14 days. Electron microscopy revealed spherical nanoparticles with a bimodal size distribution (10 nm and 75–100 nm) and a visible polysaccharide shell (5–9 nm), confirming effective stabilization. X-ray photoelectron spectroscopy confirmed the presence of metallic gold (Au^0^) and Au^1+^. NMR analysis indicated the oxidation of disulfide groups to sulfonic acid during synthesis. The nanoparticles demonstrated a high negative zeta-potential of −53.9 mV, attributable to the polyanionic alginate corona, ensuring strong electrostatic stabilization. This work establishes sulfur-modified alginic acid as an efficient platform for the rapid synthesis of stable, hybrid nanoparticles for potential use in catalysis and biomedicine.

## 1. Introduction

Among the promising “bottom-up” methods for synthesizing gold nanoparticles, a special place is occupied by the “green” approach, which uses environmentally friendly reducing agents and stabilizers of natural origin, which eliminates the need for toxic chemicals. The key components used are: plant extracts, microorganisms, biopolymers (proteins, peptides and polysaccharides) [1,2,3]. These methods make it possible to obtain nanoparticles of various shapes and sizes under mild conditions, in aqueous solutions, and with minimal energy consumption. The gold nanoparticles obtained in this way have high biocompatibility and are promising for use in catalysis and biomedicine as biosensors, drug delivery agents, and for the diagnosis and therapy of various diseases [4,5,6].

Numerous studies have explored the synthesis of gold nanoparticles (AuNPs) using polysaccharides, as well as their surface decoration with these biopolymers [7,8,9]. Surface modification of AuNPs with polysaccharides significantly alters their properties and substantially expands their range of applications. Polysaccharides form a steric and/or electrostatic barrier on the surface of the nanoparticles, which prevents their aggregation (clumping) in physiological solutions (for example, in salts) and when the pH changes [10,11]. A polysaccharide coating masks the inorganic gold core, reducing its cytotoxicity and immunogenicity [11]. Such masking can also contribute to an increase in the circulation time of nanoparticles in the bloodstream. Certain polysaccharides (dextran, hyaluronic acid, chitosan) are bioactive ligands that are recognized by specific cell receptors, which can be used for effective nanoparticle targeting [10,12,13]. The polysaccharide shell can also be covalently linked with drugs, contrast agents, and diagnostic labels, thus enabling simultaneous diagnosis and treatment of diseases [14,15].

Of particular interest among polysaccharides is alginic acid (AA), due to its availability and wide range of applications, primarily in medicine [16,17,18,19,20,21]. Several reports describe the synthesis of AuNPs using sodium alginate. For example, a facile, one-pot synthesis of gold nanoparticles (AuNPs) is achieved using an H_2_O_2_/alginate system under neutral conditions and at room temperature [22]. This method employs oxidized alginate, generated in situ, to reduce gold ions and stabilize the resulting AuNPs. Moreover, the possibility of one-step preparation of alginate-stabilized AuNPs using a microwave-induced plasma-liquid process (MWPLP) has been demonstrated [23]. A “green” synthesis strategy for metallic NPs in vitro has been developed using an alginate gel matrix, which serves as an artificial cell membrane for capturing metal-binding peptides/proteins responsible for nanoparticle formation [24].

The literature presents examples of development of diverse materials based on disulfide cross-linked alginic acid, typically positioned as drug delivery vehicles [25,26,27,28,29,30,31,32]. However, we have not found examples of their application as reagent and stabilizer in the synthesis of AuNPs.

Following our previous work [33] in synthesizing and stabilizing AuNPs with thiolated hyaluronic acid, we hypothesized that sulfur-modified alginic acid could serve as an equally effective yet functionally distinct biopolymer ligand for AuNPs. This approach is expected to endow the nanoparticles with new physicochemical and biological properties. It has been shown that a small degree of alginate modification with sulfur provides a significant increase in the rate of metal nanoparticle synthesis and the formation of stable hybrid organic–inorganic materials with unique morphology. For their characterization, methods such as electron microscopy, photon cross-correlation spectroscopy, X-ray photoelectron spectroscopy, NMR and ζ-potential measurement were employed.

## 2. Results

### 2.1. AA-AuNPs Synthesis

**Figure 1:**
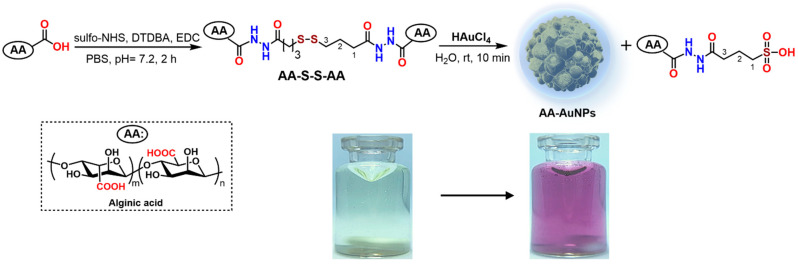
Disulfide cross-linked alginate reductive synthesis of the stable and polysaccharide in situ modified AuNP (AA-AuNPs).

At the initial stage, the synthesis of the –S–S– derivative of alginic acid **1** was carried out by reacting the polysaccharide with dithiodibutyric acid dihydrazide (DTDBA) in the presence of sulfo-NHS and the dehydrating agent N-methyl-3-(3-dimethylaminopropyl)carbodiimide hydrochloride (EDC*HCl). As a result, AA–S–S–AA with a degree of substitution of ~4.2% per monosaccharide unit was obtained (Figure 1) (determined by NMR). AA–S–S–AA was purified by dialysis. It was then reacted with HAuCl_4_ at a mass ratio of [AA–S–S–AA]:[HAuCl_4_] = 1:0.15 in bidistilled water with intensive stirring for 10 min at room temperature (20–22 °C). During this time, the color of the reaction mixture turned purple. The reaction carried out in the presence of sodium alginate at a same mass ratio of 1:0.15 for 2 h at room temperature did not result in visible formation of AuNPs.

### 2.2. NMR Spectroscopy of Disulfide Cross-Linked Alginate

**Figure 2:**
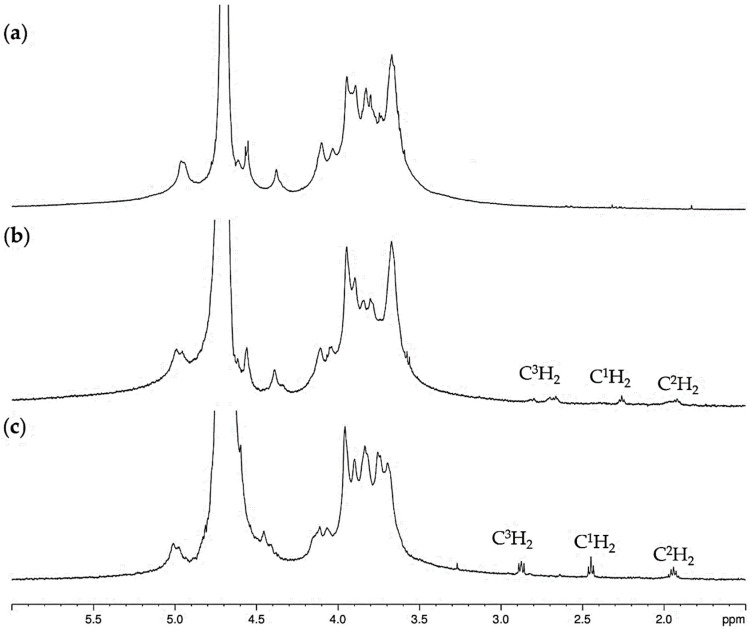
^1^H NMR spectra of compounds in D_2_O: (**a**) AA; (**b**) AA–S–S–AA; (**c**) result of the reaction of AA–S–S–AA with HAuCl_4_.

The ^1^H NMR spectrum of AA–S–S–AA (Figure 2b and Figure S6) displayed signals assignable to the methylene protons at the disulfide bond (–C^3^H_2_–S–S–) at δ_H_ 2.66 ppm, the C^1^H_2_ group at δ_H_ 2.26 ppm, and the C2 hydrogen atoms at δ_H_ 1.92 ppm.

It turned out that during the reaction, the disulfide group in compound AA–S–S–AA undergoes significant changes, resulting in the formation of sulfonic acid, similarly to what occurred in thiolated hyaluronic acid [33]. Analysis of the ^1^H NMR spectra of the AA–S–S–AA/HAuCl_4_ reaction mixture indicated the emergence of a new set of low-field signals (1.94 ppm, 2.45 ppm, and 2.87 ppm) corresponding to three methylene groups (Figure 2c, Figures S7 and S8). The multiplicity of the downfield signal at δ_H_ 2.87 (C^3^H_2_ protons) was consistent with an AA’BB’ system for four CH_2_–CH_2_ protons (J_AB_ = 6.3 Hz, J_AB’_ = 9.2 Hz) [34], which indicates the formation a symmetric –SO_3_H group that imposes constraints on the conformational mobility of the hydrocarbon chain. These findings demonstrate that the formation of alginate-coated AuNPs involves the oxidation of the S–S group to a sulfonic acid, as depicted in Figure 1.

### 2.3. AA-AuNP Characterization

#### 2.3.1. Storage Stability of AA-AuNPs

The formation of gold nanoparticles was observed using UV-Vis spectroscopy. The resulting sample was characterized by a surface plasmon resonance (SPR) peak with an absorption maximum at 539 nm. This λ_max_ value is typical for spherical gold nanoparticles with a size of several tens of nanometers [35,36].

**Figure 3:**
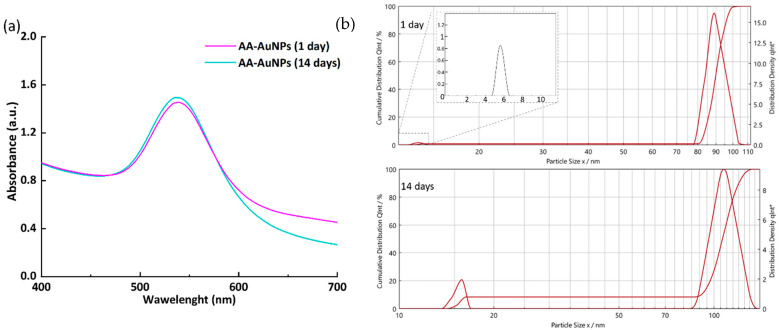
(**a**) UV-Vis spectra of AA-AuNPs; (**b**) PCCS of AA-AuNPs.

Observation of the sample over 14 days showed good stability of the nanoparticles in solution. The position of the absorption peak remained virtually unchanged (λ_max_ = 538 nm, Figure 3), indicating the absence of aggregation, sedimentation, or changes in particle size. After 14 days, the colloidal solution remained transparent and retained its characteristic purple color without the formation of visible sediment.

Time-dependent particle size analysis (Figure 3b, Figures S1 and S2) indicated two key changes over 14 days: (1) the population of smallest particles grew in average diameter from 4–7 nm to 14–17 nm, with an increase in its peak intensity, and (2) the larger size population shifted from 78–115 nm to 85–135 nm. The shifts are consistent with the onset of aggregation. However, the maintenance of a relatively narrow, bimodal distribution points to the comparative stability of the synthesized AuNPs.

Thus, the sulfur-modified alginate, acting as a stabilizing agent, effectively prevents the coalescence of gold nanoparticles, ensuring the colloidal stability of the system.

#### 2.3.2. Morphology of AA-AuNPs

**Figure 4:**
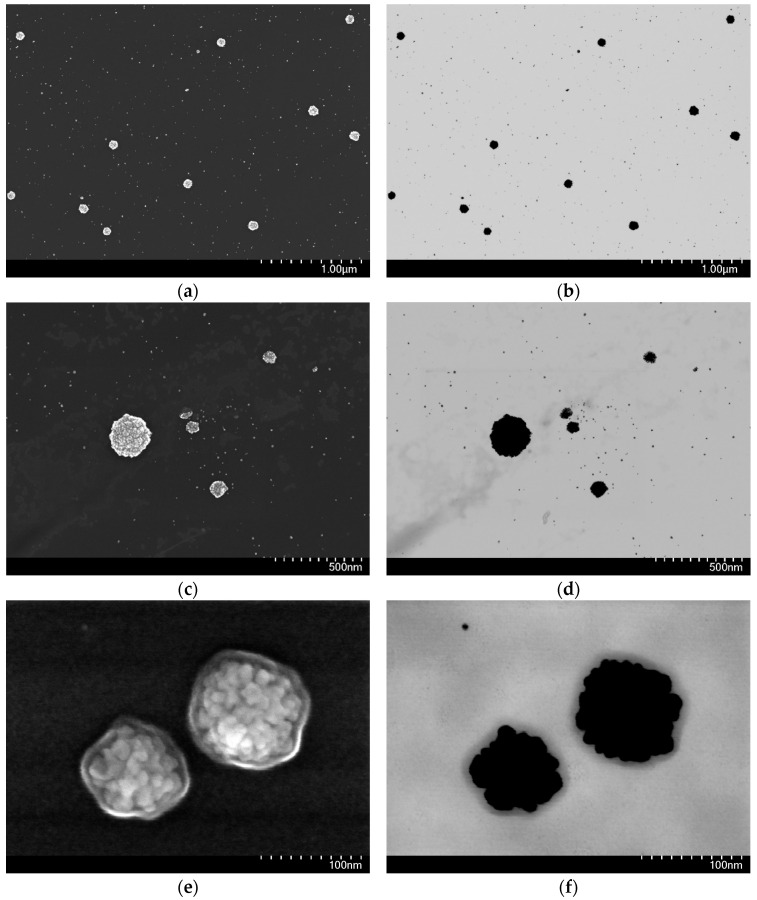
BF-STEM and SEM images of AA-AuNPs; microscopy characterization of precipitates at ×35,000 (**a**,**b**), ×60,000 (**c**,**d**), ×350,000 (**e**,**f**) magnifications (corresponding micrometer and nanometer scale bars shown on each image).

The morphology of nanoparticles was studied using scanning electron microscopy (SEM) and bright-field scanning transmission electron microscopy (BF-STEM). Figure 4 shows the morphology of AA-AuNPs. SEM analysis confirmed that the synthesized AA-AuNPs functionalized with the alginic acid derivative possess predominantly spherical morphology Figures S3 and S4). In Figure 4a–d and Figure S5, it can be seen that the system contains both small particles with sizes of about 10 nm, which apparently form larger aggregates with a diameter of 75–100 nm, correlating with the PCCS data. Figure 4e,f clearly shows a polysaccharide shell with a non-uniform thickness ranging from 5 to 9 nm, indicating the effective stabilizing role of the alginate coating, preventing particle aggregation and ensuring their colloidal stability.

#### 2.3.3. X-Ray Photoelectron Spectroscopy

**Figure 5:**
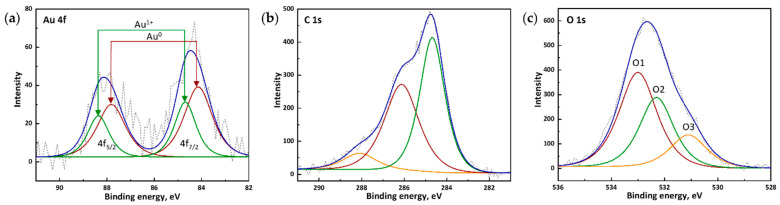
XPS spectra of AA-AuNPs: deconvolution peaks of (**a**) Au 4f, (**b**) C 1s and (**c**) O 1s (Short dash line—experimental data, blue line—summary of deconvolution lines).

During XPS analysis, the results of which are presented in Figure 5, it was shown that the AA-AuNPs spectrum is characterized by two main peaks, Au 4f_7/2_ and Au 4f_5/2_, with characteristic spin-orbit splitting values of about 3.67 eV and a peak intensity ratio of 1.33 [37,38,39,40]. The Au peak at about 84.1 eV corresponds to Au^0^ [37,38,39,40], while the line at higher energy (84.7 eV) indicates the presence of Au^1+^ [41]. The [Au^0^]: [Au^1+^] ratio was 62:37 (Table 1).

The presence of carbon on the nanoparticle surface is associated with the organic ligand. The main peak at around 284.8 eV corresponds to C–C and C–H bonds of the polysaccharide [42,43,44]. Peaks at 286.1 and 288.1 eV indicate oxygen-containing functional groups (C–O, C=O, COOH) [42,43,44,45], belonging to alginate.

Oxygen O1s is represented by three components: O1 533.0 (51.7%), O2 532.3 (33.8%), O3 531.1 (14.5%). The O1 peak at 533.0 eV can be attributed to the hydroxyl group [45,46]. The O2 component (532.3 eV) appears to correspond to oxygen atoms of the carboxyl group or to titanium hydroxide from the substrate. The O3 signal may belong to titanium oxide present on the metal substrate [47].

#### 2.3.4. ζ-Potential Measurements

The ζ-potential of the synthesized nanoparticles was measured in aqueous medium (pH ~5–6, 25.0 °C) to evaluate their colloidal stability and the nature of the stabilizing layer. As expected for a system stabilized by a polyanion, the AA-AuNPs sample demonstrated a high negative ζ-potential value of −53.9 ± 2.7 mV. Furthermore, the resulting sulfonic groups can act as a co-stabilizers for the gold nanoparticles.

The high negative ζ-potential value primarily indicates the formation of a dense polyanionic layer on the surface of the AuNPs. This charge is generated by the carboxyl groups of the main backbone of alginic acid, which, under the measurement conditions (pH ~5–6), are in a deprotonated form (–COO^−^) [48]. The flexible polymer chains of alginate form a “corona” in solution, providing both steric and electrostatic stabilization.

The absolute value of the ζ-potential (−53.9 mV), significantly exceeding the ±30 mV threshold [49], indicates the formation of an exceptionally stable colloidal system. The combination of strong electrostatic repulsion and the steric barrier created by the polymer chains effectively prevents particle aggregation under physiological conditions.

## 3. Discussion

Disulfide-crosslinked alginic acid systems represent a versatile and clinically relevant class of biomaterials [50,51]. The present study demonstrates a “green” synthetic approach for the preparation of polysaccharide-coated gold nanoparticles (AA-AuNPs) using disulfide-crosslinked alginic acid (AA–S–S–AA) as a dual-function reagent.

The known method for synthesizing S–S cross-linked alginic acid involves direct thiolation of alginate with 3-mercaptopropionic acid (achieving 1–3% DS) followed by controlled oxidative crosslinking to form disulfide bonds [50]. An alternative approach employs functionalizing alginate with cysteines modified by an enzyme-labile thiol protection group. Subsequent deprotection using penicillin G acylase (PGA) generates free thiols on-demand, enabling hydrogel cross-linking **via** thiol-reactive linkers and the formation of intermolecular disulfide bonds [51,52]. In our study, we represent the single step synthesis of AA–S–S–AA via EDC/sulfo-NHS-mediated coupling with dithiodibutyric acid dihydrazide (DTDBA) which resulted in a degree of substitution of 4.2%.

Alginic acid (or sodium alginate) is a natural biopolymer containing numerous free carboxyl (–COO^−^) and hydroxyl (–OH) groups. This provides the opportunity for the synthesis of gold nanoparticles (AuNPs) due to its ability to both reduce Au^3+^ to Au^0^ and stabilize the resulting nanoparticles [53]. Alginate-based methods, particularly solvothermal and microwave plasma processes, offer environmentally friendly, efficient synthesis with good size control, high stability, and potential for biomedical applications [22,23,24,53,54,55]. The synthesis of gold nanoparticles using S–S cross-linked AA involves harnessing the reducing properties of thiol groups within modified alginate polymers. This approach combines the structural stability provided by alginate biopolymers with the chemical reactivity of disulfide bonds, offering a biosustainable alternative to harsh reducing agents. The presence of a disulfide bridge in the molecule makes a significant contribution to the Au^3+^ reduction process. NMR analysis revealed that during the reaction, the disulfide group (–S–S–) undergoes oxidation to form sulfonic acid (–SO_3_H). We suggest that Au^3+^ here can be reduced both by the action of the disulfide S–S group with the participation of H_2_O [56,57] and by H_2_O_2_ generated during the spontaneous reduction of Au^3+^ ions (from HAuCl_4_) to Au^1+^ ions and Au^0^ nanoparticles [41]—or by molecular oxygen. The oxidation of disulfide bonds to sulfonic acid groups during interaction with gold ions represents a distinctive feature of this synthetic approach, paralleling observations made with thiolated hyaluronic acid [33]. Our findings suggest that the mechanism of gold nanoparticle formation induced by sulfur-containing biopolymers warrants more detailed investigation.

SEM and BF-STEM analysis revealed that obtained AA-AuNPs exhibit predominantly spherical morphology with a bimodal size distribution characterized by small particles (~10 nm) that form larger aggregates (75–100 nm), stabilized by a polysaccharide shell with non-uniform thickness (5–9 nm). The observation of a bimodal size distribution with small particles reflects complex nucleation and growth dynamics consistent with literature on alginate-stabilized nanoparticle synthesis [23,53]. The formation of larger aggregates (75–100 nm) from smaller primary particles is a well-known phenomenon in polysaccharide-stabilized nanoparticle systems. According to current understanding, alginate chains provide micelle-like cavities that initially limit particle growth, leading to formation of smaller primary units that subsequently associate into larger, more stable assemblies through controlled aggregation [23,53]. The non-uniform shell thickness is particularly significant and reflects the dynamic nature of polysaccharide adsorption on metal surfaces. The stabilization mechanism operates through two pathways: (i) steric stabilization provided by the physical polysaccharide barrier preventing direct contact between particles, and (ii) electrostatic stabilization from carboxylate groups (–COO^−^) of alginate chains creating negative surface charge [58]. The SPR band position and its stability over 14 days of observation (λ_max_ shifted only to 538 nm), as well as high negative charge (−53.9 mV), confirm the colloidal stability of the synthesized nanoparticles, which is also characteristic of alginate-coated AuNP [23].

Future studies should emphasize long-term storage validation, biological medium compatibility assessment, and mechanistic investigation of the aggregation-stabilization equilibrium to fully elucidate the practical applicability of these AA-AuNP systems for biomedical applications.

## 4. Materials and Methods

*General Information.* The following reagents were used for the synthesis: the sodium alginate were supplied from Shanghai Aladdin Biochemical Technology Co., Ltd. (Shanghai, China), N-hydroxysulfosuccinimide sodium salt (sulfo-NHS) (>98%, TCI, Tokio, Japan), 4,4′-Dithiodibutyric acid (95%, Sigma-Aldrich, Chemie GmbH, Steinheim, Germany), N-methyl-3-(3-dimethylaminopropyl)carbodiimide hydrochloride (EDC*HCl, 98%, abcr GmbH, Karlsruhe, Germany). 4,4′-Dithiodibutyric acid dihydrazide (DTDBA) was obtained by the method [59].

^1^H and ^13^C NMR spectra were acquired on a Bruker AVANCE-500 spectrometer (Bruker, Rheinstetten, Germany;) (500.17 and 125.78 MHz, respectively) using D_2_O as the solvent. Samples were prepared in standard 5 mm NMR tubes. Chemical shifts (δ) are reported in ppm relative to tetramethylsilane (TMS). Standard Bruker pulse sequences were employed to record one- and two-dimensional (COSY ^1^H–^1^H) spectra.

*Synthesis of –S–S–cross-linked alginic acid.* Disulfide-crosslinked alginate (AA–S–S–AA) was obtained by reacting activated carboxyl groups of the polymer with dithiodibutyric acid dihydrazide (DTDBA) according to the method described for hyaluronic acid [60].

200 mg of sodium alginate were dissolved in 20 mL of phosphate-buffered saline (PBS, 0.1 M, pH 7.2) with constant stirring to obtain a 1% (*w*/*v*) solution. To activate the carboxyl groups, 13.6 mg (0.063 mmol) of sulfo-N-hydroxysuccinimide sodium salt (sulfo-NHS) were added to the alginate solution and stirred until completely dissolved. Then, 24.0 mg (0.125 mmol) of N-methyl-3-(3-dimethylaminopropyl)carbodiimide hydrochloride (EDC*HCl), pre-dissolved in 1 mL of distilled water, were quickly added to the reaction mixture. The mixture was stirred for 20 min at room temperature to complete the activation. To the activated alginate solution, 27.8 mg (0.104 mmol) of DTDBA dissolved in 2 mL of PBS (0.1 M, pH 8.5) were added. To achieve the target degree of functionalization of ~4.2%, the molar ratio between the activated carboxyl groups of alginate and the hydrazide groups of DTDBA was maintained at 1.2:1. The crosslinking reaction was carried out for 2 h at room temperature with constant stirring. The resulting product was purified by dialysis against distilled water (with water changes every 6–8 h) for 48 h.

*Synthesis of AA-AuNPs.* The synthesis of AuNPs was carried out by reacting AA–S–S–AA with HAuCl_4_. For this, an aqueous solution of HAuCl_4_ (with a concentration of 10 mg/mL) was added with intensive stirring to an aqueous solution of AA–S–S–AA with a concentration of 5.1 mg/mL at a mass ratio of [AA–S–S–AA]:[HAuCl_4_] = 1:0.15, respectively. The reaction was carried out at room temperature with intensive stirring for 10 min.

*Characterization of AuNPs.* The sample microstructures were characterized using a Hitachi Regulus 8220 electron microscope (Hitachi High-Tech Corporation, Tokyo, Japan), operating in both scanning electron microscopy (SEM) and bright-field scanning transmission electron microscopy (BF-STEM) modes. The samples were prepared by deposition onto 3 mm carbon-coated copper grids. Images were acquired in transmission electron mode using a 30 kV accelerating voltage.

XPS analysis was performed on a JEOL JPS 9010MX spectrometer (JEOL, Tokyo, Japan) using an Mg Kα X-ray source, with the analytical chamber maintained under ultra-high vacuum (<7 × 10^−8^ Pa). Samples were prepared by depositing AA-AuNP solutions onto titanium plates (Grade 4) and allowing the solvent to evaporate. Survey spectra (0–1100 eV) were recorded with a pass energy of 50 eV and a step size of 0.5 eV. All binding energies were referenced by calibrating the C 1s peak to 284.8 eV. Peak analysis, quantitative elemental composition, and spectral deconvolution of the high-resolution spectra using Voigt functions were carried out with the JEOL SpecSurf software (v. 1.9.0).

Optical properties of AA-AuNPs solutions were studied using the UV–Vis spectrophotometer UV-1800 (Shimadzu, Tokio, Japan).

Particle size distribution was analyzed using Photon Cross-correlation Spectroscopy (PCCS) on a NanoPhox instrument (Sympatec, Clausthal-Zellerfeld, Germany). Measurements were conducted in triplicate at 25 °C, and data were processed with the PAQXOS 4.2 software. Prior to sample analysis, the instrument’s accuracy was verified using Nanosphere™ Size Standards (23 ± 2, 100, and 510 ± 7 nm; ThermoFisher Scientific, Waltham, MA, USA).

The ζ-potential of the AA-AuNPs was determined via optical heterodyning technique, using a Zetasizer Nano ZS instrument (Malvern Instruments Ltd., Malvern, UK).

## 5. Conclusions

This work presents a “green” method for synthesizing polysaccharide-coated gold nanoparticles (AA-AuNPs) using a disulfide-crosslinked alginic acid (AA) as a dual-function reagent. A slight modification of alginate with sulfur-containing groups (~4.2%) drastically accelerated Au^3+^ reduction, enabling AA-AuNP formation within 10 min at room temperature.

The resulting AA-AuNPs feature a bimodal size distribution and maintain excellent colloidal stability for over two weeks. A key mechanistic insight, supported by NMR data, is the oxidation of the disulfide bridge to sulfonic acid groups during nanoparticle formation.

The sulfur-modified alginate acts as both a reducing agent and a stabilizer. It forms a protective polyanionic layer around the nanoparticles, providing a combination of steric and electrostatic stabilization, as evidenced by the high negative ζ-potential (−53.9 mV).

The proposed method is a simple, environmentally friendly, and effective approach to producing stable hybrid organic–inorganic nanomaterials with potential applications in biomedicine and catalysis.

## Figures and Tables

**Table 1:** Fitting parameters of the Au 4f spectrum for AA-AuNPs.

Sample	Au	Binding Energy (eV) 4f_7/2_	Binding Energy (eV) 4f_5/2_	Rel. Area (%)
AA-AuNPs	Au^3+^	-	-	-
	Au^1+^	84.7	88.4	37.23 ± 0.89
	Au^0^	84.1	87.8	62.77 ± 1.49

## Data Availability

The datasets generated during and/or analyzed during the current study are available from the corresponding author on reasonable request.

## Supplementary Materials

The following supporting information can be downloaded at: https://www.mdpi.com/article/10.3390/molecules30244750/s1. Figure S1. PCCS of AA-AuNPs (1 day); Figure S2. PCCS of AA-AuNPs (14 days); Figure S3. BF-STEM (a,b) and SEM (c) images of AA-AuNPs; Figure S4. SEM (a) and BF-STEM (b) images of AA-AuNPs; Figure S5. SEM (a) and BF-STEM (b) images of AA-AuNPs; Figure S6. NMR 1H spectra of AA–S–S–AA; Figure S7. NMR 1H spectra of the reaction mixture of AA–S–S–AA with HAuCl4; Figure S8. 2D NMR COSY HH spectra the reaction mixture of AA–S–S–AA with HAuCl4.
