# Supplementary material for: Reductive Synthesis of Stable, Polysaccharide in Situ-Modified Gold Nanoparticles Using Disulfide Cross-Linked Alginate
Source: Molecules. 2025 Dec 12;30(24):4750. doi: 10.3390/molecules30244750 (PMC12735437; doi:10.3390/molecules30244750)

Reductive Synthesis of Stable, Polysaccharide in Situ-Modified Gold Nanoparticles Using Disulfide Cross-Linked Alginate

Lyudmila V. Parfenova [1,*], Eliza I. Alibaeva [1], Guzel U. Gil'fanova [1], Zulfiya R. Galimshina [1], Ekaterina S. Mescheryakova [1], Leonard M. Khalilov [1], Semen N. Sergeev [2], Nikita V. Penkov [3] and Baoqiang Li [4]

[1] Institute of Petrochemistry and Catalysis, Ufa Federal Research Center, Russian Academy of Sciences, Prospekt Oktyabrya, 141, 450075 Ufa, Russia; a.e_l_i_z_a@mail.ru (E.I.A.); gilfanova.guzel@gmail.com (G.U.G.); lollipiip@mail.ru (Z.R.G.); katusha2974@gmail.com (E.S.M.);khalilovlm@gmail.com (L.M.K.)

[2] Department of Materials Science and Physics of Metals, Institute of Technology and Materials, Ufa University of Science and Technology, 12 Karl Marx Street, 450008 Ufa, Russia; nikocem17@gmail.com

[3] Institute of Cell Biophysics of the Russian Academy of Sciences, Federal Research Center "Pushchino Scientific Center for Biological Research of the Russian Academy of Sciences", Institutskaya 3, 142290 Pushchino, Russia; nvpenkov@rambler.ru

[4] State Key Laboratory of Urban Water Resource and Environment, Institute for Advanced Ceramics, Harbin Institute of Technology, Harbin 150001, China; libq@hit.edu.cn

* Correspondence: luda_parfenova@mail.ru

Supporting Information

Figure S1. PCCS of **AA-AuNPs** (1 day) 2
Figure S2. PCCS of **AA-AuNPs** (14 days) 2
Figure S3. BF-STEM and SEM images of **AA-AuNPs**. 3
Figure S4. BF-STEM and SEM images of **AA-AuNPs**. 4
Figure S5. BF-STEM and SEM images of **AA-AuNPs** 5
Figure S6. NMR ^{1}H spectra of **AA–S–S–AA** 6
Figure S7. NMR ^{1}H spectra of the reaction mixture of **AA–S–S–AA** with $HAuCl_4$ 7
Figure S8. 2D NMR COSY HH spectra the reaction mixture of **AA–S–S–AA** with $HAuCl_4$ 8

Figure S1. PCCS of **AA-AuNPs** (1 day)

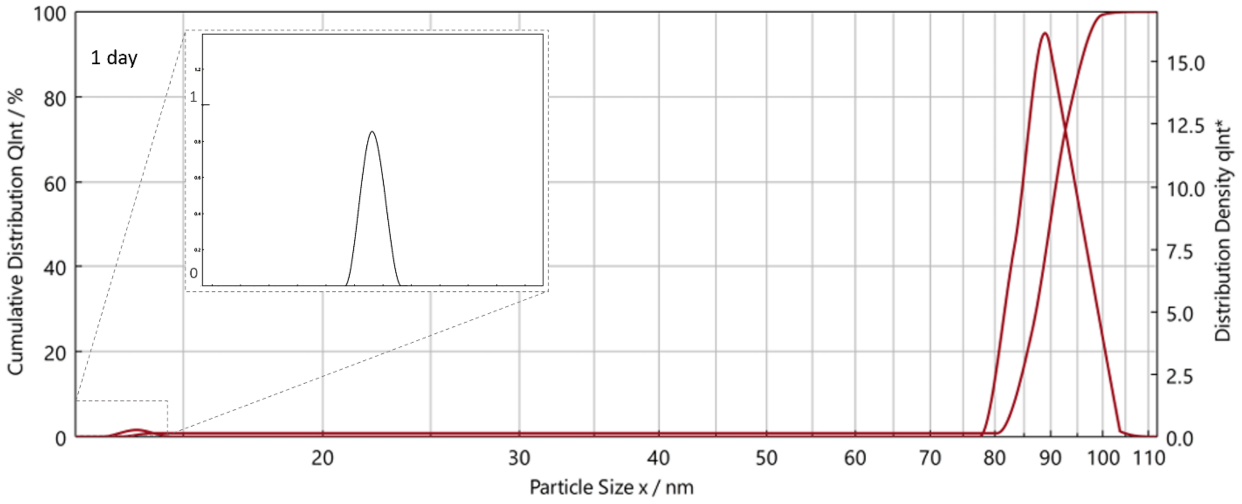

Figure S2. PCCS of **AA-AuNPs** (14 days)

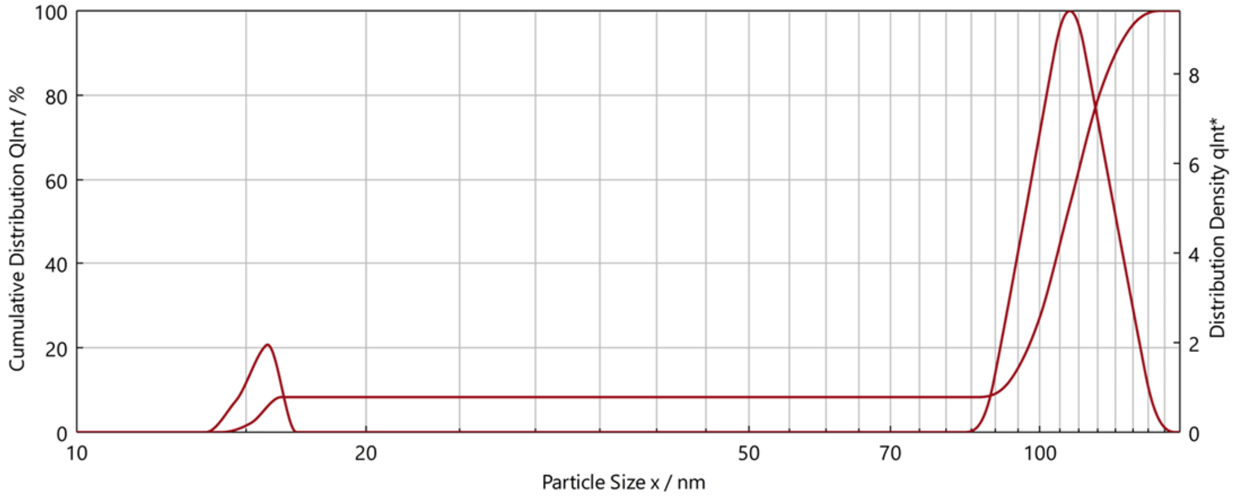

Figure S3. BF-STEM (**a**,**b**) and SEM (**c**) images of **AA-AuNPs**; microscopy characterization of precipitates at ×35 000 magnifications (corresponding micrometer and nanometer scale bars shown on each image).

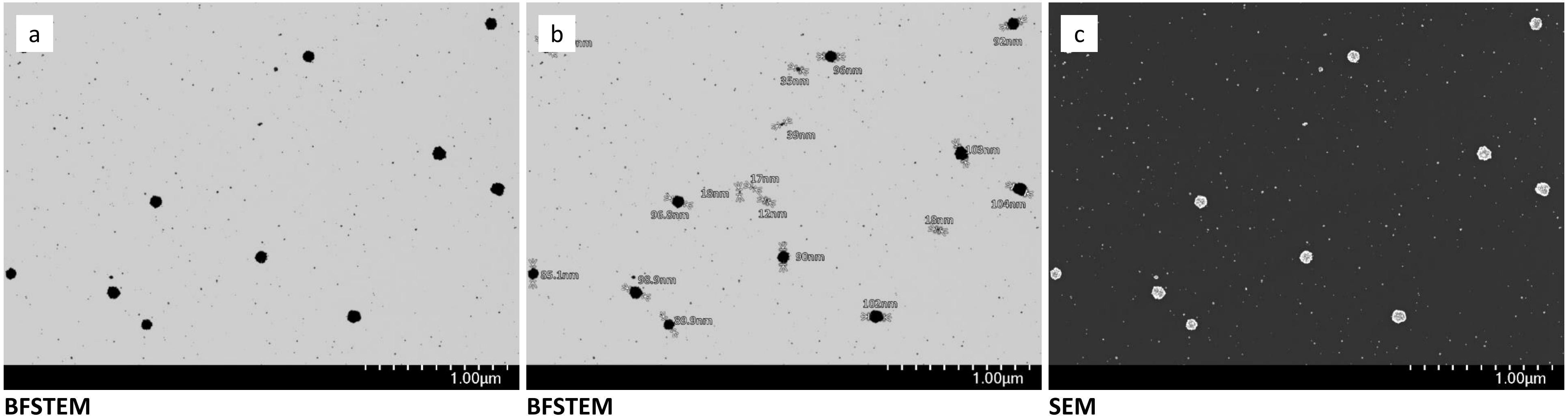

Figure S4. SEM (**a**) and BF-STEM (**b**) images of **AA-AuNPs** with the dimensions of the protrusions indicated (corresponding micrometer and nanometer scale bars shown on each image).

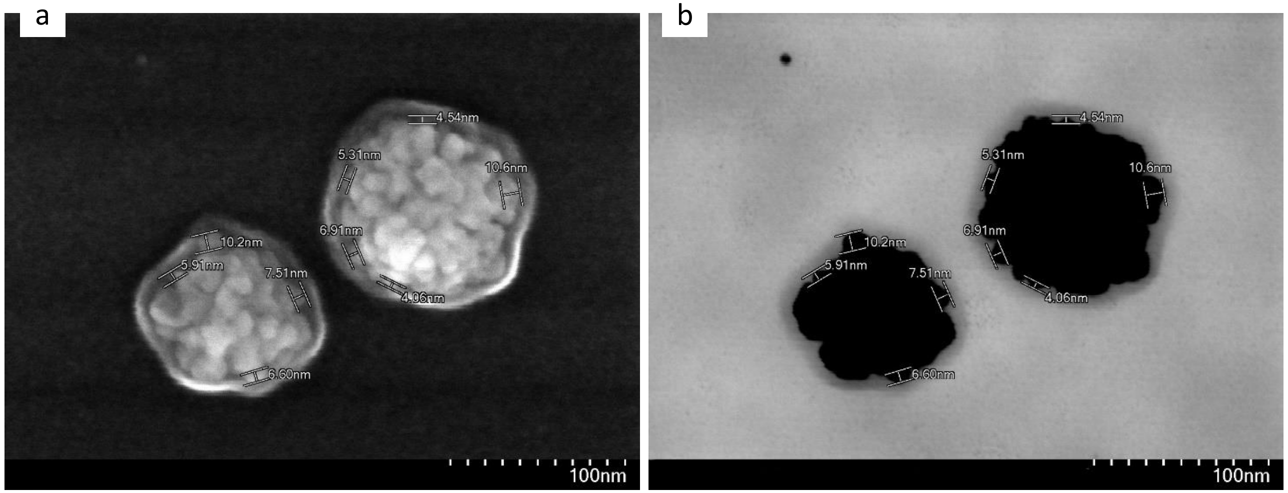

SEM **BFSTEM**

Figure S5. SEM (**a**) and BF-STEM (**b**) images of **AA-AuNPs** with their sizes indicated (corresponding micrometer and nanometer scale bars shown on each image).

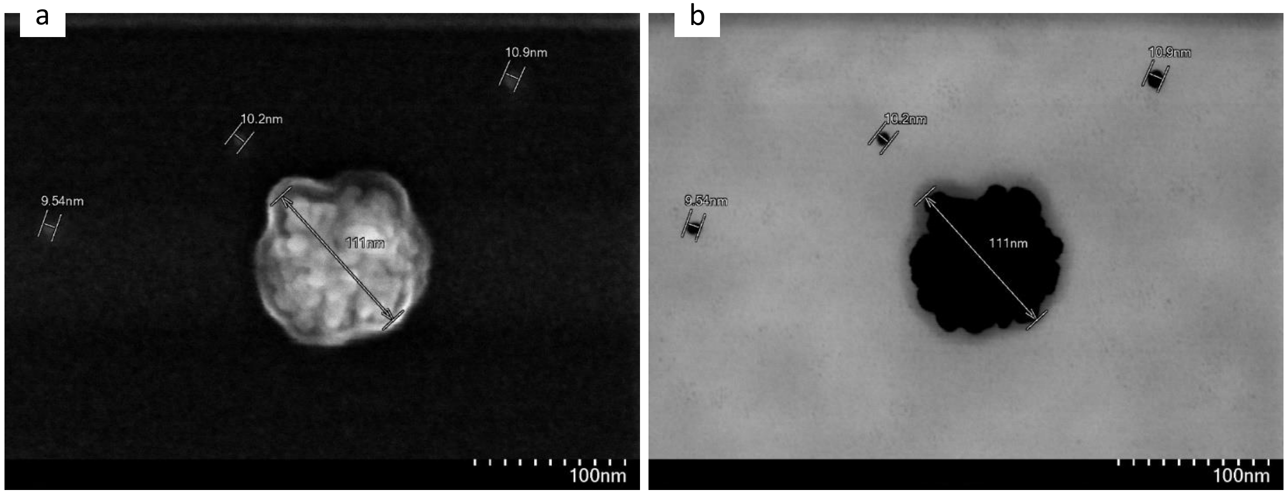

SEM **BFSTEM**

Figure S6. NMR ^{1}H spectra of **AA–S–S–AA**

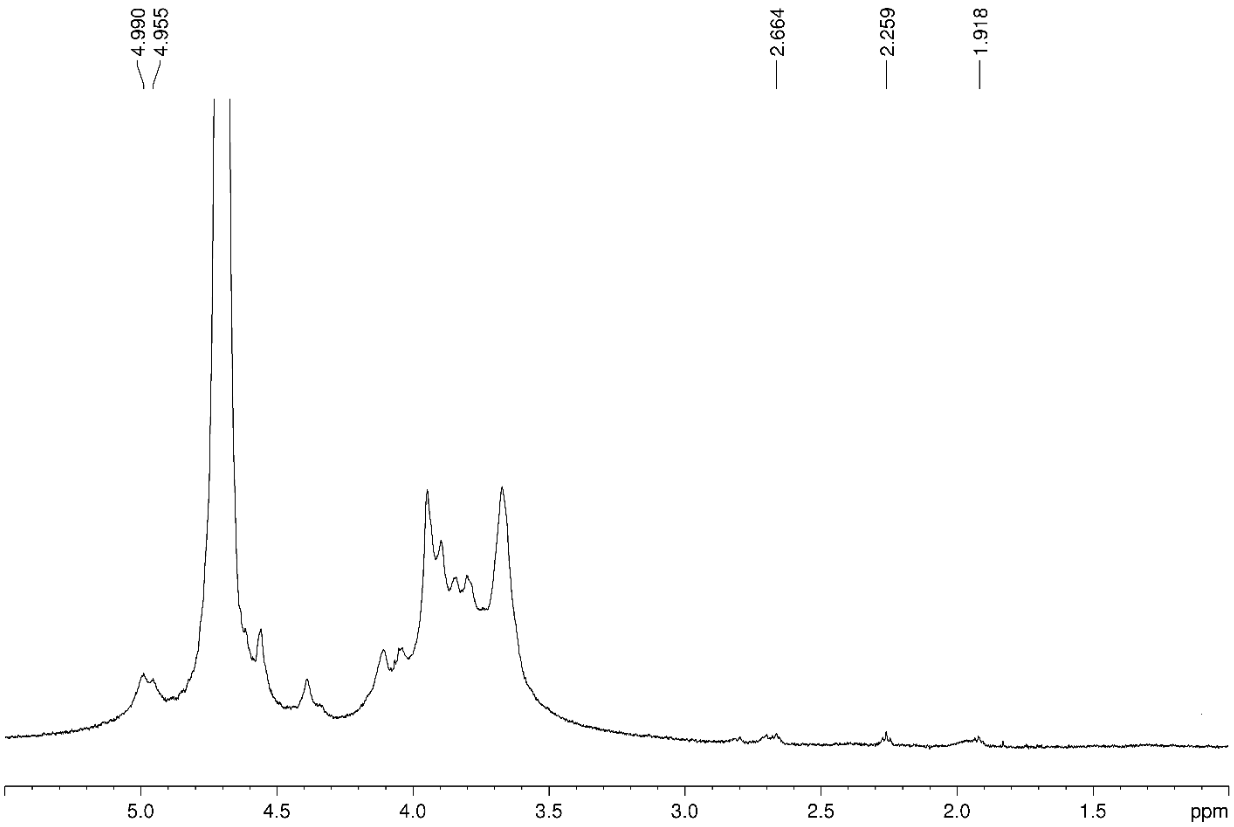

Figure S7. NMR ^{1}H spectra of the reaction mixture of **AA–S–S–AA** with $HAuCl_4$

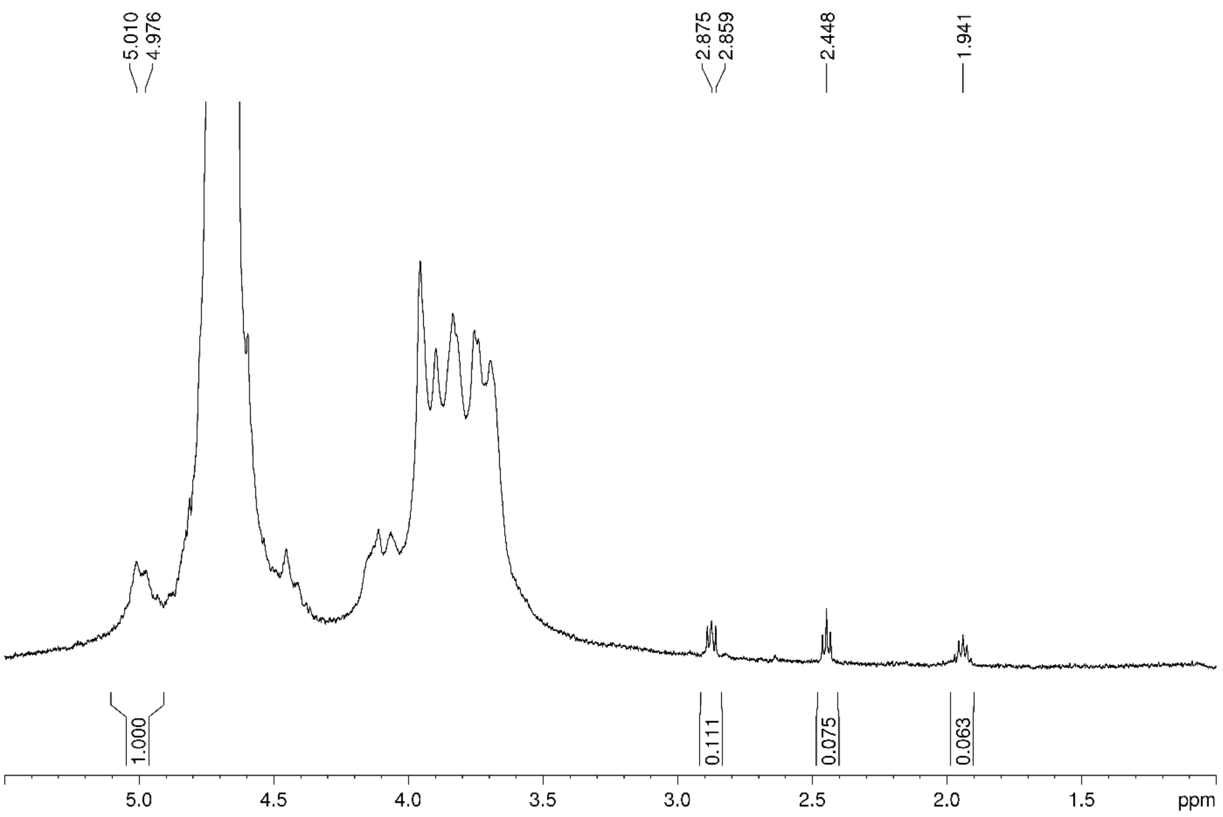

Figure S8. 2D NMR COSY HH spectra the reaction mixture of **AA–S–S–AA** with $HAuCl_4$

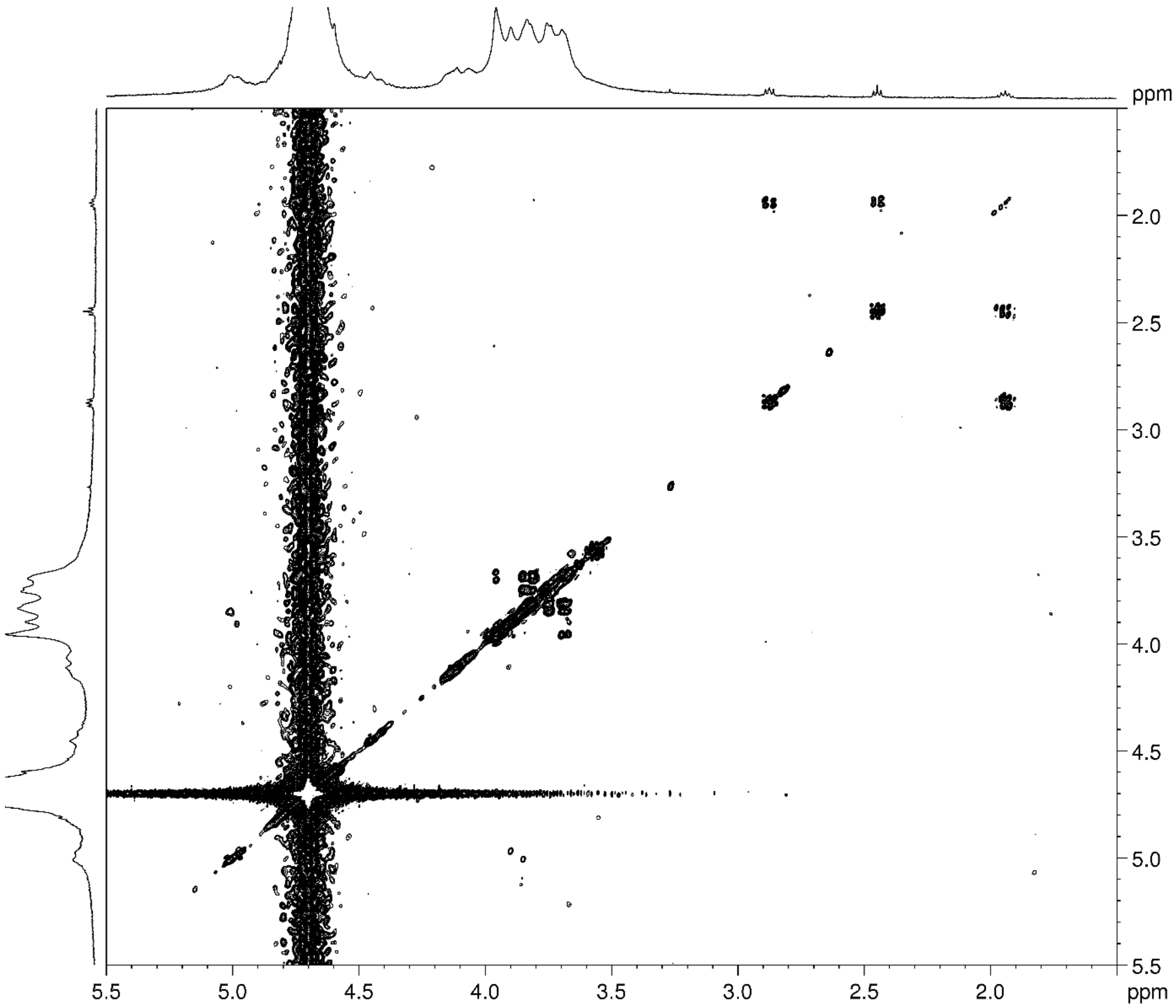

Supplement: Supplementary file 1 [file molecules-30-04750-s001.zip › molecules-4007912-supplementary.pdf]
